# Supplementary material for: Structural insights into GABAA receptor potentiation by Quaalude
Source: Nat Commun. 2024 Jun 19;15:5244. doi: 10.1038/s41467-024-49471-y (PMC11187190; doi:10.1038/s41467-024-49471-y)
Supplement: Supplementary file 1 — Supplementary Information [file 41467_2024_49471_MOESM1_ESM.pdf]

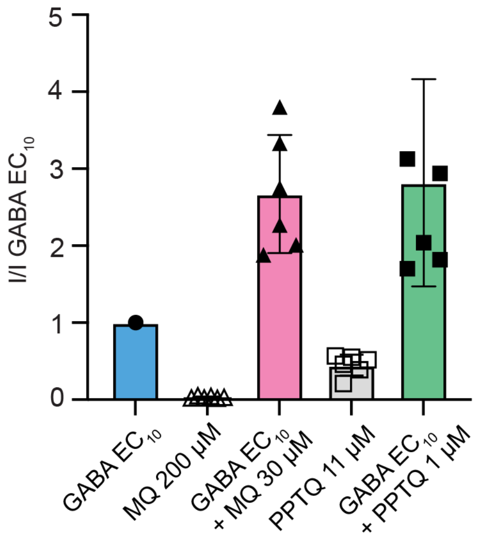

Supplementary Figure 1. Bar graph showing replicates of the electrophysiology recordings from Figure 1a. Results are shown as a mean response±S.D.; n=6 recordings from independent cells.

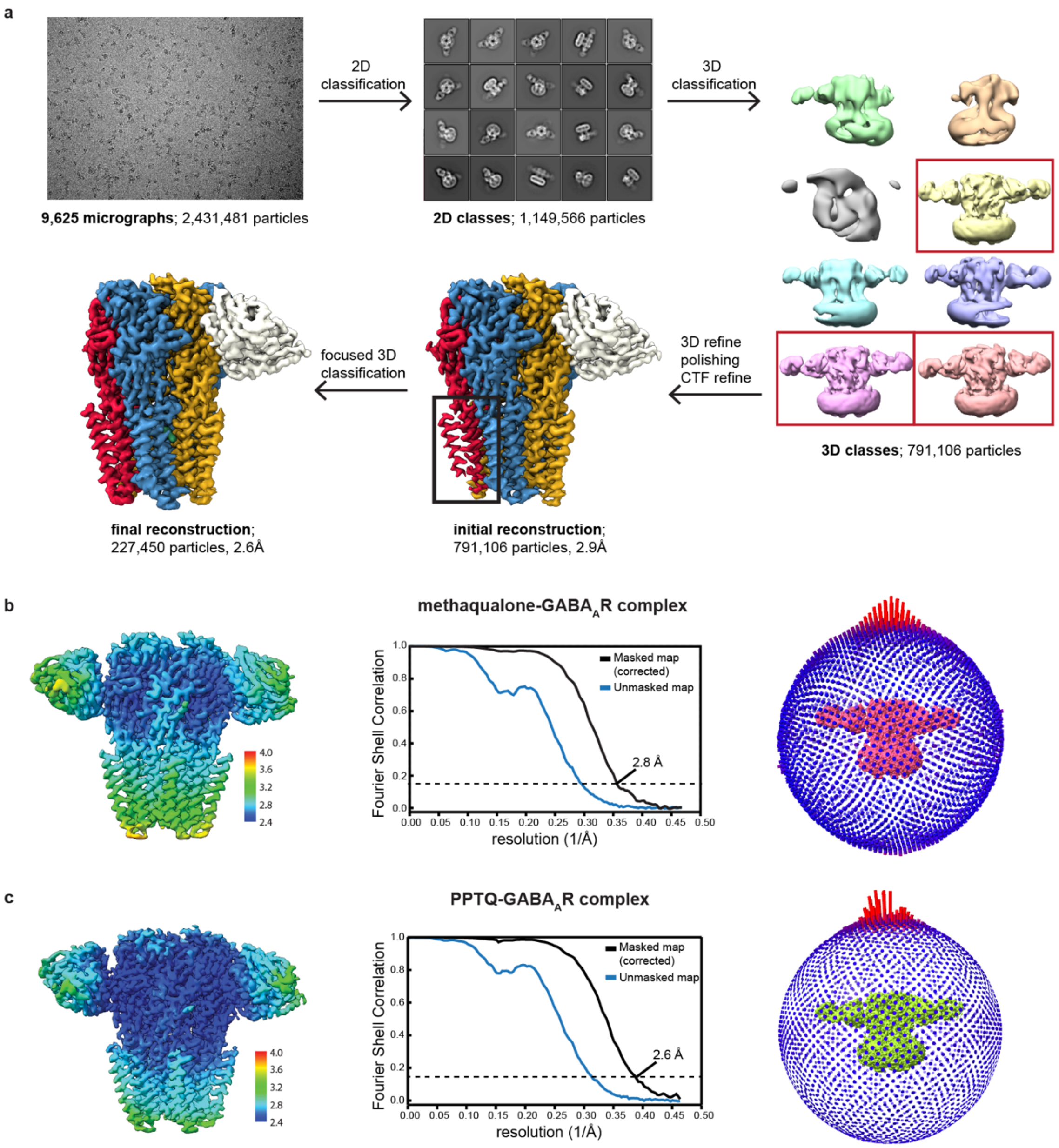

Supplementary Figure 2. Cryo-EM processing workflow. a Data processing and map reconstruction for PPTQ dataset. **b** and **c** Local resolution maps, FSC curves and angular distribution of particles used in the final reconstruction for **b** methaqualone-GABA$_A$R complex, **c** PPTQ-GABA$_A$R complex.

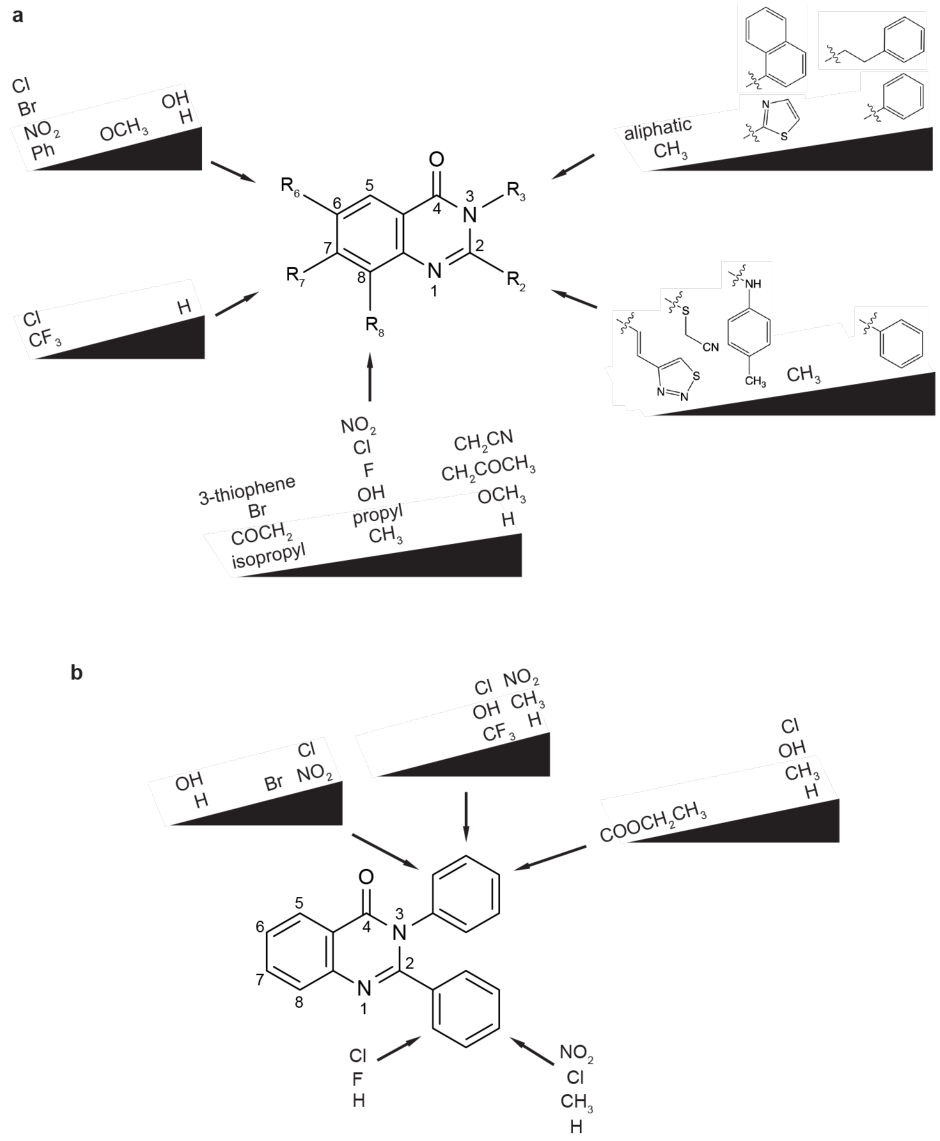

Supplementary Figure 3. SAR trends for quinazolinones as $GABA_A$ receptor PAMs. Schematic representation of the effects mediated by various substituents introduced at **a** the quinazolinone core[1,2] and **b** the phenyl rings of the quinazolinone analog 2,3-diphenylquinazolin-4(*3H*)-one (PPQ)[1] on $GABA_A$ receptor PAM potency.

Supplementary discussion

The structural information presented in our work supports previous extensive structure-activity relationship (SAR) studies of quinazolinones as $GABA_A$ receptor modulators. These included series of analogs with substituents at the 2-, 3-, 6-, 7- and 8- positions of the quinazolinone core[1,2], as well as analogs comprising substitutions at the 2- and 3-position phenyl ring of 2,3-diphenylquinazolin-4(*3H*)-one (PPQ)[1]. The SAR trends observed in these studies are schematically presented in **Supplementary Fig. 3**.

As highlighted in the main text, substitution of the 2-methyl in methaqualone to a phenyl in PPQ and PPTQ yields a substantial increase in PAM potency due to increased number of interactions with the neighboring amino acids. In contrast, introduction of heteroaromatic and polar substituents in this position is unfavorable and yields analogs characterized by considerably lower PAM activity than methaqualone[1]. Interestingly, PPQ analogs comprising small substituents in the *ortho*- (chloro, fluoro) and/or *para*- (nitro, chloro, methyl) positions of the 2-phenyl ring do not significantly impact PAM potencies compared to PPQ itself[1].

Whereas quinazolinone analogs comprising methyl or other aliphatic groups in the 3-position are either completely devoid of or display very low PAM potencies, introduction of aromatic groups like *o*-tolyl or *p*-tolyl (in methaqualone and PPTQ, respectively), phenyl in PPQ or phenethyl in this position substantially increases PAM activity[1]. Analogs comprising heteroaromatic (thiazol-2-yl) or bulkier aromatic ring systems exhibit intermediate potencies[1]. In contrast to the negligible effects of small substitutions in the 2-phenyl ring, the substitution pattern on the 3-phenyl ring in PPQ appears to be an important determinant of PAM potency[1]. While introduction of smaller substituents, such as methyl (PPTQ), hydroxy and chloro in the *para*-position of this ring does not significantly impact the PAM potency, a bulkier ethyl acetate substituent in this position results in a ~50-fold decreased PAM potency. Relatively small substituents (methyl, trifluoro, hydroxy, nitro, chloro) introduced in the *meta*-position of the 3-phenyl ring all have modest effects on PAM potency. In contrast, introduction of nitro, chloro or bromo in the *ortho*-position of the 3-phenyl ring in PPQ leads to substantial (5-10 fold) increases in PAM potency, whereas the presence of a hydroxy group in this position does not significantly change PAM activity. Based on the quinazolinone-bound structures, the *o*-substituent in the 3-phenyl ring would lay in close proximity to residues on either the principal β or the complementary α subunit side, depending on its favored position in reference to the quinazolinone ring, thus creating additional interactions. Interestingly, this increase in PAM potency is not seen for in a 3-(2,6-dichlorophenyl)-containing PPQ analog, suggesting that only one of the two *ortho*-substituents is beneficial for PAM activity, and that this 3-(2,6-dichlorophenyl) analog in its binding mode cannot adopt the spatial orientation needed to harvest this beneficial effect from this *ortho*-substituent.

As also outlined in the main text, introduction of substitutions in the 6-, 7- and 8-positions of the quinazolinone core influences PAM potency differently, but overall substituents in these positions are not beneficial for $GABA_A$ receptor activity[2]. Whereas the PAM potency displayed by PPQ is retained in its 6-hydroxy-substituted analog, introduction of methoxy, chloro, bromo, nitro and phenyl groups yield analogs with substantially reduced PAM potencies. 7-chloro or 7-trifluoromethyl-substituted PPQ analogs also exhibit substantially reduced PAM potencies. Finally, whereas the PAM potency of PPQ is retained in analogs comprising methoxy, 2-oxypropyl and acetonitrile substituents in the 8-position, the corresponding methyl, *n*-propyl, hydroxy, fluoro, chloro and nitro analogs exhibit 5-9-fold lower potencies than PPQ, and introduction of larger (3-thiophene, bromo), branched aliphatic (isopropyl) but also small (acetyl) substituents in this position reduce PAM potency even farther.

These results largely support our observations in the structural data. Substituents to the quinazolinone scaffold that face the entrance of the TMD β/α binding pocket cannot be too bulky nor polar due to the hydrophobic nature of the surrounding environment. However, addition of aromatic moieties at the 2- and 3- positions of the quinazolinone core positively impacts PAM potency by establishing additional interactions with binding pocket residues. The quinazolinone cores of methaqualone and PPTQ align well in the structures and therefore it is safe to assume that this is the most stable position of the quinazolinone core in reference to the protein. Finally, since the 6- 7- and 8-positions in the quinazolinone core orient deeply, making tight interactions with the M2 helices, anything but the smallest substitutions in these positions would be expected to be unfavorable for modulator binding to this site.

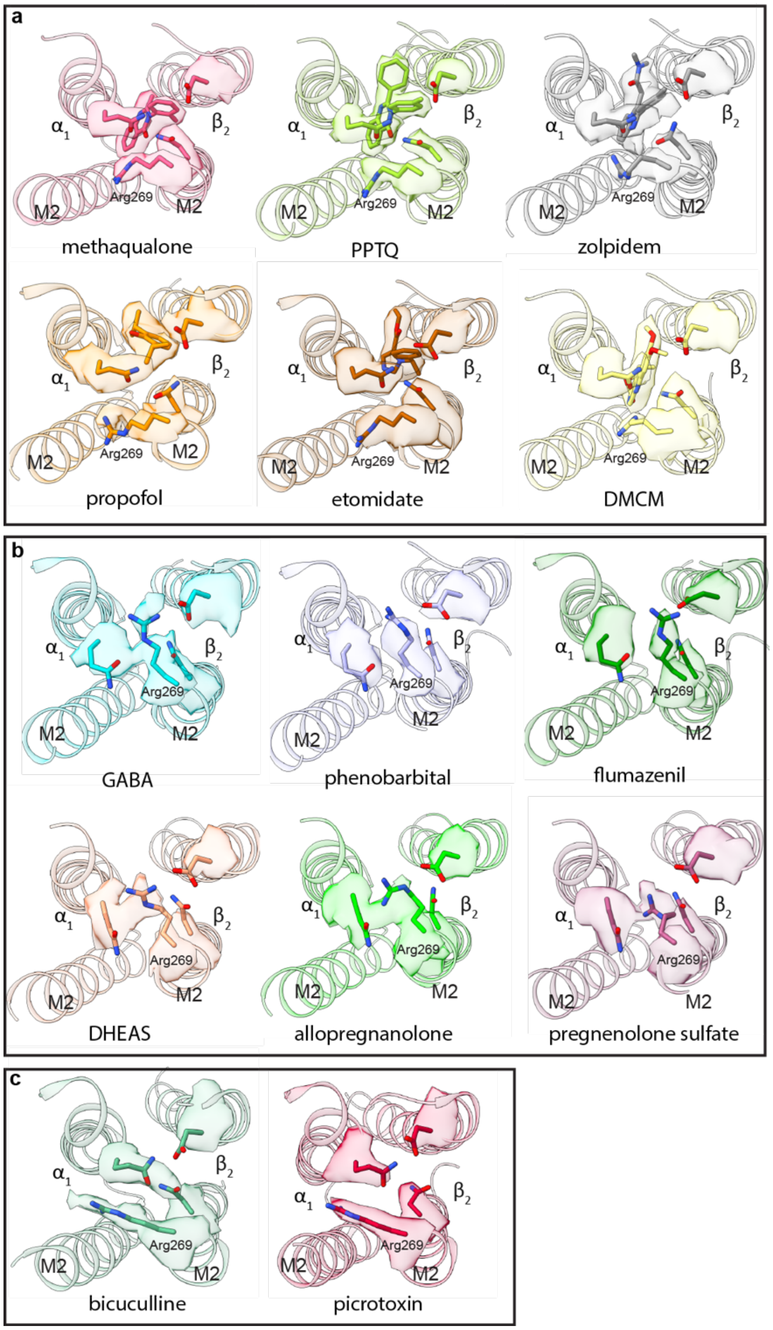

Supplementary Figure 4. Comparisons of βR269 position in different structures. a Arginine position between the M2 helices in structures with occupied β2/α1 binding site; PDB IDs: zolpidem-8DD2, propofol-6X3T, etomidate-6X3V, DMCM-8DD3. **b** Arginine position above the β2/α1 binding site in structures where this site is not occupied; PDB IDs: GABA-6X3Z, phenobarbital-6X3W, flumazenil-6X3U, DHEAS-8SID, allopregnanolone-8SI9, pregnenolone sulfate-8SGO **c** Arginine position between M1 and M2 helices of the α_1 subunit in structures in a resting-like state; PDB IDs: bicuculline-6X3S, picrotoxin-6HUG.

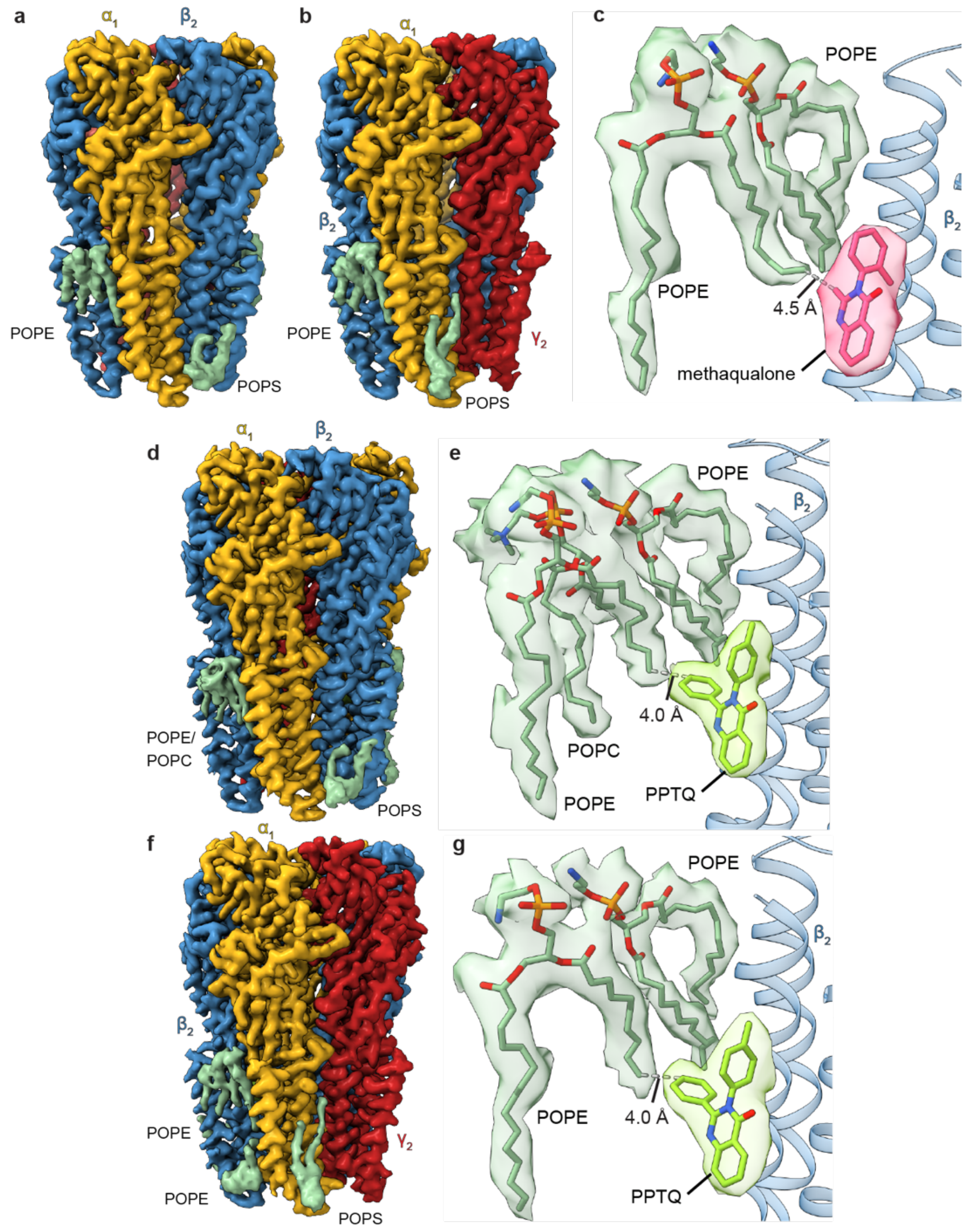

Supplementary Figure 5. Lipid distribution in quinazolinone-bound complexes. a and **b** global distribution of lipids for methaqualone structure. **c** lipids adjacent to the binding site of methaqualone. **d** and **f** global distribution of lipids for PPTQ structure. **e** and **g** lipids adjacent to the PPTQ binding sites. **c,e,g** α_1 subunit helices were hidden for clarity.

	GABA+PPTQ (EMD-43485) (PDB 8VRN)	GABA+methaqualone (EMD-43475) (PDB 8VQY)	
**Data collection and processing**		Dataset 1	Dataset 2
Magnification	81,000×	81,000×	81,000×
Voltage (kV)	300	300	300
Electron exposure (e^{-}/Å^2)	50	50	50
Number of frames	52	50	50
Defocus range (µm)	-0.8 to -2.2	-0.8 to –1.8	-0.8 to –2.0
Processing pixel size (Å)	1.079	1.07	1.069
Corrected pixel size (Å)	1.07	-	-
Symmetry imposed	C1	C1	C1
Number of images	9,625	9,813	7,824
Initial particle images (no.)	2,431,481	1,554,293	1,378,687
Final particle images (no.)	227,450	189,247	
Map resolution (Å)	2.57	2.82	
FSC threshold	0.143	0.143	
Map resolution range (Å)	2.24-5.49	2.41-5.87	
**Refinement**			
Initial model used (PDB code)	6X3V	PPTQ model	
Model resolution (Å)	2.57	2.89	
FSC threshold	0.5	0.5	
Map sharpening B-factor (Å)	-30	-30	
Model composition			
Non-hydrogen atoms	17,865	17,785	
Protein residues	2,121	2,121	
Ligand	43	41	
B factors (Å^2)			
Protein	52	84	
Ligand	82	118	
R.m.s. deviations			
Bond lengths (Å)	0.006	0.004	
Bond angles (°)	0.785	0.664	
Validation			
MolProbity score	1.54 (99th percentile)	1.43 (100th percentile)	
Clashscore	5.2 (99th percentile)	3.89 (100th percentile)	
Poor rotamers (%)	0	0	
Ramachandran plot			
Favored (%)	96.1	96.2	
Allowed (%)	3.9	3.8	
Disallowed (%)	0	0	

Supplementary Table 1. Cryo-EM data collection, refinement and validation statistics for GABA+PPTQ and GABA+methaqualone complexes.

Mutation	Methaqualone	PPTQ
α1β2^{M286W}γ2	Decreased PAM activity[3].	Elimination of direct activation and pronounced decrease in PAM activity[1].
α1β2^{M286A}γ2	Not tested.	Elimination of direct activation and decreased PAM activity[1].
α1^{M236W}β2γ2	Pronounced increase in direct activation[3].	Pronounced increase in direct activation and decreased PAM activity[1].
α1^{M236A}β2γ2	Not tested.	No significant difference in both direct activation and PAM activity[1].
α6β2^{N265S}δ	Elimination of PAM activity[3].	Not tested.
α6β1^{S265N}δ	Rescued PAM activity[3].	Not tested.
α1β2^{N265M}γ2	Almost eliminated PAM activity[3].	Elimination of both direct activation and PAM activity[1].
α1^{T265A}β2γ2	Decreased PAM activity*.	Decreased PAM and agonist activities*.
α1^{L269A}β2γ2	Decreased PAM activity*.	Decreased PAM and agonist activities*.

Supplementary Table 2. Summary of effects of mutations in β2/α1 binding pocket residues on methaqualone and PPTQ activities. *this study

References

1. Madjroh, N., Rie, E., Bundgaard, C., Cecilia, P. & Jensen, A. A. Functional properties and mechanism of action of PPTQ , an allosteric agonist and low nanomolar positive allosteric modulator at GABA A receptors. *Biochem. Pharmacol.* **147**, 153–169 (2018).
2. Wang, P. F., Jensen, A. A. & Bunch, L. From Methaqualone and Beyond: Structure - Activity Relationship of 6-, 7-, and 8-Substituted 2,3-Diphenyl-quinazolin-4(3H)-ones and in Silico Prediction of Putative Binding Modes of Quinazolin-4(3H)-ones as Positive Allosteric Modulators of GABAA Receptor. *ACS Chem. Neurosci.* **11**, 4362–4375 (2020).
3. Hammer, H. *et al.* A multifaceted GABAA receptor modulator: Functional properties and mechanism of action of the sedative-hypnotic and recreational drug methaqualone (Quaalude). *Mol. Pharmacol.* **88**, 401–420 (2015).
