# Supplementary material for: Structural insights into GABAA receptor potentiation by Quaalude
Source: Nat Commun. 2024 Jun 19;15:5244. doi: 10.1038/s41467-024-49471-y (PMC11187190; doi:10.1038/s41467-024-49471-y)
Supplement: Supplementary file 3 — Description of Additional Supplementary Files [file 41467_2024_49471_MOESM3_ESM.pdf]

Description of Additional Supplementary Files

File Name: Supplementary Movie 1
Description: Global 360° view of the $GABA_A$ receptor complex with GABA and methaqualone, map and model. α1 subunits are colored yellow, β2 subunits are colored blue, γ2 subunit is colored red, Fab fragments are colored white, GABA is colored salmon, methaqualone is colored pink, lipids are colored green, and sugars are colored tan.

File Name: Supplementary Movie 2
Description: Close-up rocking view on the methaqualone TMD binding site at the β2/α1 subunit interface, map and model. α1 subunit is colored yellow, β2 subunit is colored blue, and methaqualone is colored pink.

File Name: Supplementary Movie 3
Description: Close-up rocking view of the methaqualone ECD binding site at the α1/γ2 subunit interface, map and model. α1 subunit is colored yellow, γ2 subunit is colored red, and methaqualone is colored pink.

File Name: Supplementary Movie 4
Description: Global 360° view of the $GABA_A$ receptor complex with GABA and PPTQ, map and model. α1 subunits are colored yellow, β2 subunits are colored blue, γ2 subunit is colored red, fab fragments are colored white, GABA is colored salmon, PPTQ is colored bright green, lipids are colored green, and sugars are colored tan.

File Name: Supplementary Movie 5
Description: Close-up rocking view on the PPTQ TMD binding site at the β2/α1 subunit interface, map and model. α1 subunit is colored yellow, β2 subunit is colored blue, PPTQ is colored bright green.
